# Development and validation of a nomogram for predicting prostate cancer in patients with PSA ≤ 20 ng/mL at initial biopsy

**DOI:** 10.1097/MD.0000000000028196

**Published:** 2021-12-17

**Authors:** Qiang Wu, Fanglong Li, Xiaotao Yin, Jiangping Gao, Xu Zhang

**Affiliations:** a Department of Graduate Administration, Chinese PLA General Hospital, Beijing, China; b Department of Urology, Huhhot First Hospital, Huhhot, China; c Department of Urology, Chinese PLA 980th Hospital, Shijiazhuang, China; d Senior Department of Urology, the Third Medical Center of PLA General Hospital, Beijing, China.

**Keywords:** biopsy, diagnosis, nomogram, prostate-specific antigen, prostatic cancer

## Abstract

The aim of this study was to construct a nomogram for predicting prostate cancer (PCa) in patients with PSA ≤ 20 ng/mL at initial biopsy.

The patients with PSA ≤ 20 ng/mL who underwent prostate biopsy were retrospectively included in this study. The nomogram was developed based on predictors for PCa, which were assessed by multivariable logistic regression analysis. The receiver operating characteristic curve, calibration plots and decision curve analysis (DCA) were used to evaluate the performance of the nomogram.

This retrospective study included 691 patients, who were divided into training set (505 patients) and validation set (186 patients). The nomogram was developed based on the multivariable logistic regression model, including age, total PSA, free PSA, and prostate volume. It had a high area under the curve of 0.857, and was well verified in validation set. Calibration plots and DCA further validated its discrimination and potential clinical benefits. Applying the cut-off value of 15%, our nomogram would avoid 42.5% of unnecessary biopsies while miss only 4.4% of PCa patients.

The nomogram provided high predictive accuracy for PCa in patients with PSA ≤ 20 ng/mL at initial biopsy, which could be used to avoid the unnecessary biopsies in clinical practice.

## Abstract 2

Supplemental Digital Content is available in the text

## 1 Introduction

Prostate cancer (PCa) is the most common malignancy in men worldwide, and its incidence is also rapidly increasing in China. According to the Chinese National Cancer Institute, it is estimated that there is 60,300 new cases of PCa in 2015 in China.^[1]^ The prostate biopsy is currently the standard practice for the diagnosis of PCa,^[2,3]^ whereas it could not be used as an extensive screening tool due to its medical cost and invasiveness.^[4,5]^ Therefore, it is more important for urologist to identify the patients with a high risk of PCa for further examinations in clinical practice.

Prostate-specific antigen (PSA) is the most frequently used screening test for PCa, which was first introduced into clinical use in 1980 s and promoted the detection rate of PCa significantly.^[6,7]^ But there has always been controversy over PSA screenings for men since its application. First, PSA is a prostate-specific rather than a PCa-specific marker, and some benign prostate diseases may also lead to the abnormal elevation of PSA, such as benign prostate hyperplasia (BPH) and prostatitis.^[8]^ Especially in the range of 4 to 10 ng/mL, there is significant overlap in PSA levels between BPH and PCa patients. Moreover, it is reported that PCa detection rates on initial prostate biopsy ranged between 22.8% and 42.0%, which is partially ascribed to the low specificity of PSA.^[9]^ There are still many unnecessary prostate biopsies screened by current PSA test. In addition, it is reported that patients with a PSA level ≤ 4 ng/mL still have the risk of PCa, and the detection rate may reach up to 20%.^[10,11]^

In the past few years, some predictive tools were developed to help identify the PCa risk before biopsy, such as probability table, artificial neural network, and nomogram.^[12–18]^ Comparing with other tools, the nomogram could integrate different risk factors and provide an individualized estimation of PCa probability. Besides, the nomogram can be displayed graphically and easily applied in clinical practices. However, most nomograms were constructed on European or American populations, and fewer models focused on Asian populations.

Therefore, on the basis of the large cohort of Chinese patients who underwent prostate biopsies in our medical center, we developed a nomogram that incorporates several simple information, including age, total PSA, free PSA, and prostate volume to predict the risk for PCa before biopsy.

## 2 Methods

### 2.1 Ethical approval

All the procedures followed were in accordance with the Helsinki Declaration (1964, amended in 1975, 1983, 1989, 1996, and 2000) of the World Medical Association. This study was approved by the Ethics Committee of Chinese PLA General Hospital.

### 2.2 Study design and patient population

The study was a retrospective observational study, which was complied with the STROBE statement (Strengthening the Reporting of Observational studies in Epidemiology).

**Figure 1:**
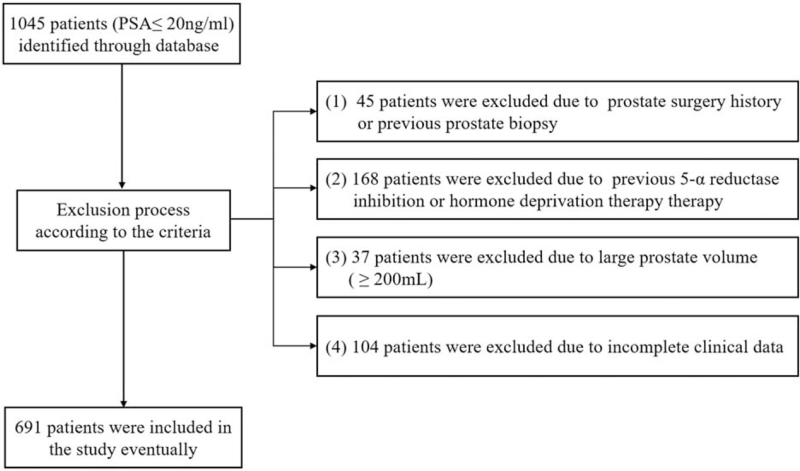
Flow chat of patient exclusion according to the criteria.

Following institutional review board approval, patients with PSA ≤ 20 ng/mL who had undergone transrectal ultrasound-guided prostate biopsy between July 2009 and March 2018 were initially collected from the Chinese PLA General Hospital. The PLA General Hospital is located in Beijing and is a large tertiary hospital, which provides medical services to people in Beijing area and surrounding provinces. The annual outpatient volume of the PLA General Hospital reaches 8 million. According to the Chinese guideline for diagnosis and treatment of PCa, prostate biopsy should be recommended for patients with the following conditions: PSA is greater than 10 ng/mL; PSA is between 4 and 10 ng/mL, but PSA density (PSAD) or free/total PSA ratio (f/t PSA) results are abnormal; Regardless of PSA level, suspicious digital rectal examination (DRE) or transrectal ultrasonography (TRUS) result. All patients who met the above criteria were recommended for a prostate biopsy. The age, total PSA (tPSA), free PSA (fPSA), prostate volume, and the prostate biopsy result including Gleason score of patients were retrospectively collected. In order to avoid the potential bias, the serum PSA test and TRUS examination were all performed in our hospital. The exclusion criteria contained: We excluded the patients who had prostate surgery history or previous prostate biopsy; Patients who had used 5-α reductase inhibition or hormone deprivation therapy; Patients with prostate volume > 200 mL; and Patients with incomplete clinical data. Overall, 691 patients were enrolled in this study, and the process of patient inclusion and exclusion is shown in Figure 1. The eligible patients were randomly divided into a training set (nomogram development, 505 cases) and a validation set (nomogram validation, 186 cases).

Serum PSA concentrations (tPSA and fPSA) were measured before DRE and TRUS by enzyme-linked immunoassay (EIA; Research & Development Systems). The width (W), length (L), and height (H) of prostate were measured on TRUS, and PV (mL) was calculated using the formula: PV = W × L × H × 0.52. TRUS was performed by experienced ultrasonologists.

### 2.3 Nomogram development

To develop a well-calibrated nomogram for predicting the risk of PCa, we performed univariable as well as multivariable logistic regression analyses to evaluate the relationship between the associated factors and PCa. First, the variables that exhibited a statistical significance (*P* < .05) in univariable logistic analysis were then included in the multivariable logistic regression analysis, which was performed to screen independent predictors for PCa. Independent predictors in multivariable logistic regression analysis were finally included in the nomogram construction.

### 2.4 Evaluating the nomogram performance

#### 2.4.1 Calibration plots

The calibration plot with bootstrapping was used to illustrate the association between the actual probability and the predicted probability, which reflected performance characteristics of the nomogram.^[19]^

#### 2.4.2 Receiver operating characteristic (ROC)

The ROC curves of the nomogram in training set and validation set were also compared and area under curve (AUC) was calculated.^[20]^ The AUC ranged from 0 to 1, with 1 indicating perfect concordance, 0.5 indicating no better than chance, and 0 indicating discordance. And the ROCs of PSA alone, f/t PSA and PSAD were also measured and their AUCs were compared with that of the nomogram model by the DeLong method respectively.^[21]^ And as a screening tool for PCa, the sensitivity of the nomogram should be about 95%.

#### 2.4.3 Decision-curve analysis

The decision curve analysis was performed to further evaluate the performance of the nomogram.^[22]^

#### 2.4.4 Internal validation

The AUC of the developed nomogram was verified in the validation set. And the ROC of nomogram was also compared with the tPSA, f/t PSA, and PSAD.

### 2.5 Statistical analysis

The Pearson's Chi-square test was used to evaluate the distribution of categorical variables, and Mann–Whitney *U* test or Student test was used for distribution of continuous variables. The univariable and multivariable logistic analysis was performed using SPSS software (version 20.0; SPSS Company, Chicago, IL). The development and evaluation of the nomogram were carried out using R software version 3.31 (R Foundation for Statistical Computing, http://www.r-project.org). In this study, all *P* values were 2-sided with statistical significance at *P* < .05.

## 3 Results

### 3.1 Patient characteristics

Among 1144 patients who underwent prostate biopsy, 691 of them were eligible and included in this study eventually. Five hundred five cases were randomly divided into training set and 186 cases into validation set. The patients’ characteristics are summarized in Table 1. The mean age of the patients was 68.92 ± 9.78 years, mean tPSA was 9.16 ± 4.82 ng/mL, mean fPSA was 1.38 ± 0.93 ng/mL, and mean prostate volume 54.67 ± 30.76 mL. Overall, 262 patients (38%) had positive biopsy results and were diagnosed with PCa. Among them, 103 patients had a Gleason score of 6, 50 of 3+4 = 7, 49 had of 4 + 3 = 7, 35 had of 8, and 25 had of 9 to 10. Age, fPSA, tPSA, and prostate volume were all significantly different between biopsy-positive and negative groups.

**Table 1:** Baseline clinical characteristics and comparison between patients with positive and negative results on prostate biopsy.

		PCa		
Variable	Total	Positive	Negative	*P*
Total patients	691	262	429	
Age, yr				
Mean ± SD	68.92 ± 9.78	71.82 ± 9.01	67.14 ± 9.82	<.001
Median	70	73	68	
IQR	62–76	66–79	61–74	
tPSA, ng/mL				
Mean ± SD	9.16 ± 4.82	10.38 ± 4.69	8.42 ± 4.76	<.001
Median	8.61	9.90	7.99	
IQR	5.53–12.40	6.73–13.80	5.10–11.40	
fPSA, ng/mL				
Mean ± SD	1.38 ± 0.93	1.30 ± 0.87	1.43 ± 0.97	.012
Median	1.21	1.19	1.25	
IQR	0.70–1.92	0.76–1.77	0.64–2.01	
Prostate volume, mL				
Mean ± SD	54.67 ± 30.76	40.36 ± 21.94	63.45 ± 32.09	<.001
Median	47.97	34.14	57.30	
IQR	31.67–71.57	24.78–49.76	40.67–80.82	
f/t PSA				
Mean ± SD	0.17 ± 0.12	0.14 ± 0.07	0.18 ± 0.14	<.001
Median	0.15	0.13	0.17	
IQR	0.10–0.21	0.09–0.16	0.12–0.24	
PSAD				
Mean ± SD	0.22 ± 0.18	0.32 ± 0.21	0.15 ± 0.11	<.001
Median	0.16	0.26	0.13	
IQR	0.10–0.28	0.16–0.43	0.08–0.19	
Gleason score (number)				
6		103		
3 + 4 = 7		50		
4 + 3 = 7		49		
8		35		
9–10		25		

IQR = interquartile range, PCa = prostate cancer, SD = standard deviation.

### 3.2 Predictors for positive biopsy results

In univariable logistic regression analysis, patient age, tPSA, fPSA, and prostate volume were significantly related to the presence of PCa in biopsy (Table 1). The significant predictors were then included in the multivariable logistic regression analysis. According to the result, patients with older age [*P* < .001, odds ratio (OR) = 1.082, 95% confidence interval (95% CI): 1.059–1.105] and elevated tPSA (*P* < .001, OR = 1.196, 95% CI: 1.137–1.258) were more likely to have positive prostate biopsy results, while patients with higher fPSA (*P* = .048, OR = 0.695, 95% CI: 0.522–0.924) and bigger prostate volume (*P* < .001, OR = 0.956, 95% CI: 0.947–0.966) were less likely to have positive results (Table 2). And the Constant listed in Table 2 was obtained by the multivariate logistic regression analysis, which represents the intercept of the equation. Eventually, the age, tPSA, fPSA, and prostate volume were all significantly related to PCa and used for construction of the nomogram.

**Table 2:** Multivariate logistic regression analysis of factors associated with PCa.

Variable	PCa			
	Coefficient	*P*	OR	95% CI
Age	0.079	<.001	1.082	1.059–1.105
tPSA	0.179	<.001	1.196	1.137–1.258
fPSA	−0.364	.048	0.695	0.522–0.924
Prostate volume	−0.045	<.001	0.956	0.947–0.966
Constant	−4.865	<.001	0.008	–

CI = confidence interval, fPSA = free PSA, OR = odds ratio, PCa = prostate cancer, tPSA = total PSA.

### 3.3 Construction of the nomogram

**Figure 2:**
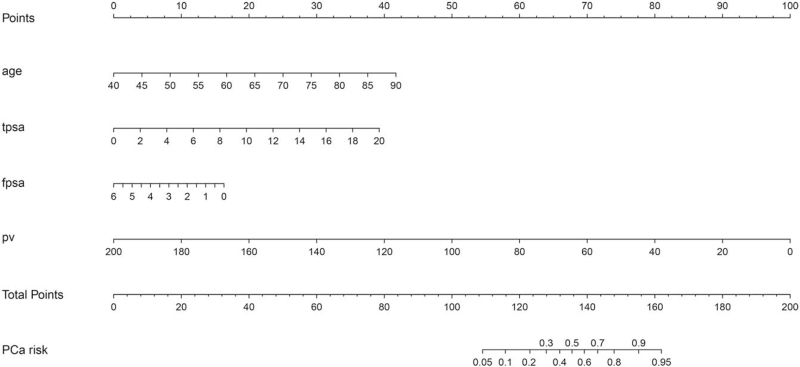
The nomogram for predicting the risk of PCa at initial systematic 12-core prostate biopsy.

Nomograms for predicting PCa (Fig. 2) were developed according to the coefficient of the four significant predictors: age, tPSA, fPSA, and prostate volume. The total point was summed up by the point of each variable (top plotting scale), then was subject to the risk for PCa (bottom plotting scale).

### 3.4 Calibration plot and discrimination of the nomogram for PCa detection in the training set

**Figure 3:**
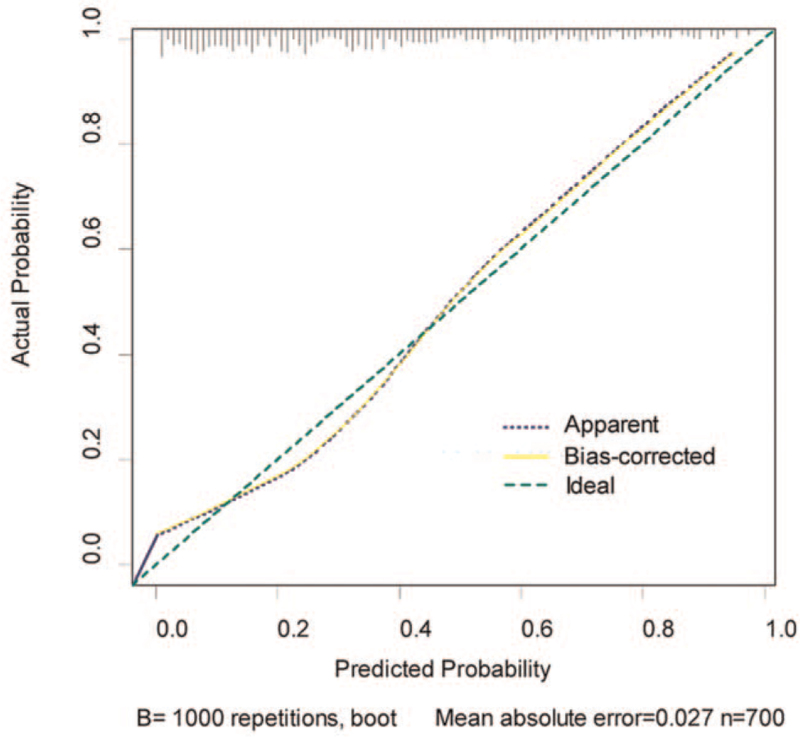
Calibration plot of the nomogram (1000 bootstrap re-samples) for predicting risk of PCa. The y-axis represents actual probability, and x-axis represents the probability estimated by nomogram (Predicted probability).

**Figure 4:**
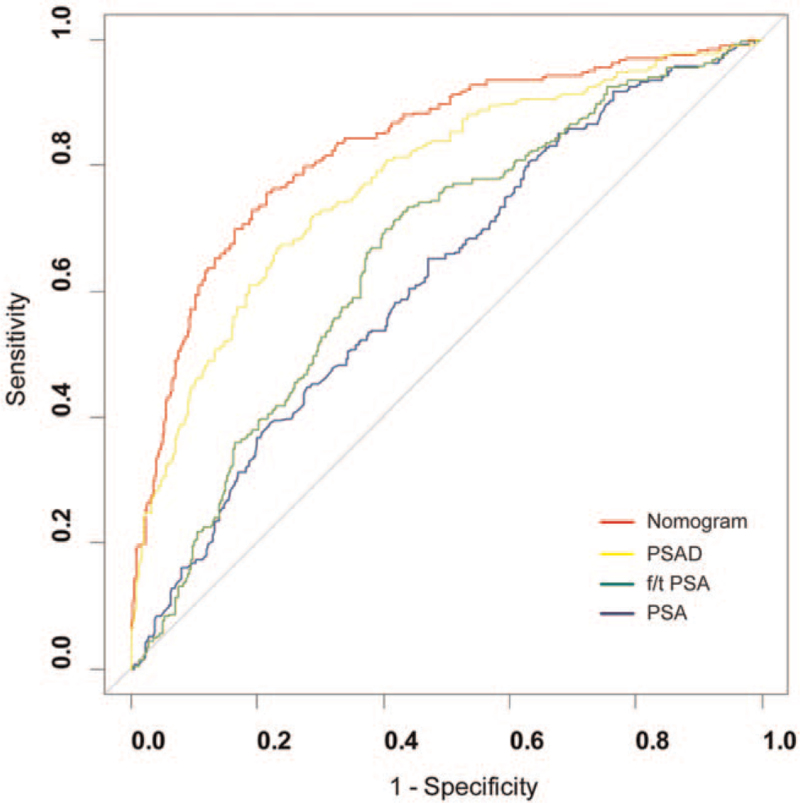
The ROC of nomogram and other parameters for predicting the risk of PCa at initial biopsy in training set.

The calibration plot with internally bootstrap sampling (n = 1000) demonstrated that bias-corrected curve was close to the ideal curve (the 45-degree line), which indicated the good calibration of our nomogram (Fig. 3). The ROC was performed to evaluate the predictive accuracy of the nomogram in the training set and validation set respectively. For predicting PCa, the AUC was 0.857 (95% CI: 0.823–0.890) in training set. And the AUC of the nomogram was significantly better than that of PSA (0.664, *P* < .001), f/t PSA (0.669, *P* < .001), or PSAD (0.801, *P* < .05) (Fig. 4, Table 3).

**Table 3:** Comparison of the AUC between the nomogram and PSA-related parameters for predicting PCa at initial biopsy.

	PCa		
	AUC	95% CI	*P* ^∗^
Nomogram	0.857	0.823–0.890	–
PSA	0.664	0.616–0.712	<.001
f/t PSA	0.669	0.621–0.717	<.001
PSAD	0.801	0.761–0.840	<.05

AUC = area under the curve, CI = confidence interval, PCa = prostate cancer, PSA = prostate-specific antigen, PSAD = PSA density.

∗ The AUC of nomogram was compared with those of PSA, f/t PSA, and PSAD, respectively.

### 3.5 Internal validation of the nomogram for PCa detection in validation set

The nomogram was validated in the 186 patients of validation set. The acceptable calibration plot result was also observed. The ROC analysis indicated that AUC of the nomogram was 0.834, which was consistent with the AUC of nomogram in training set. Compared with PSA, f/t PSA, or PSAD, the AUC of nomogram was also superior in validation set (Table 3).

### 3.6 Performance of the nomogram for PCa detection

For PCa detection nomogram, the optimal risk cut-off value was 15% with a sensitivity of 95.6%, specificity of 42.5%, positive prediction value (PPV) of 53.0%, and negative prediction value (NPV) of 93.4%. And this cutoff would avoid 42.5% of unnecessary biopsies and 25.3% of total biopsies, with missing only 4.4% of PCa patients. A direct comparison of cut-off performances relative to available alternatives was performed at a 95% sensitivity analysis of nomogram against PSA, f/t PSA, and PSAD (Table 4). And the performance of the nomogram at different cut-off value is shown in Supplementary table 1, http://links.lww.com/MD2/A747.

**Table 4:** Performance comparison of nomogram to alternative models at 95% sensitivity in the training cohort of n = 505 patients.

Method	Cut-off value	Sensitivity	Specificity	PPV	NPV	Predicted negative
Nomogram	15%	95.6%	42.5%	53.0%	93.4%	25.3%
PSA	3.31	95.1%	17.3%	43.8%	83.9%	12.3%
f/t PSA	0.055	95.0%	8.3%	39.5%	46.9%	6.3%
PSAD	0.079	95.1%	25.6%	45.9%	87.8%	16.2%

f/t PSA = free/total prostate-specific antigen, NPV = negative predictive value, PPV = positive predictive value, PSAD = PSA density.

### 3.7 Decision-curve analysis of the nomogram in clinical practice

**Figure 5:**
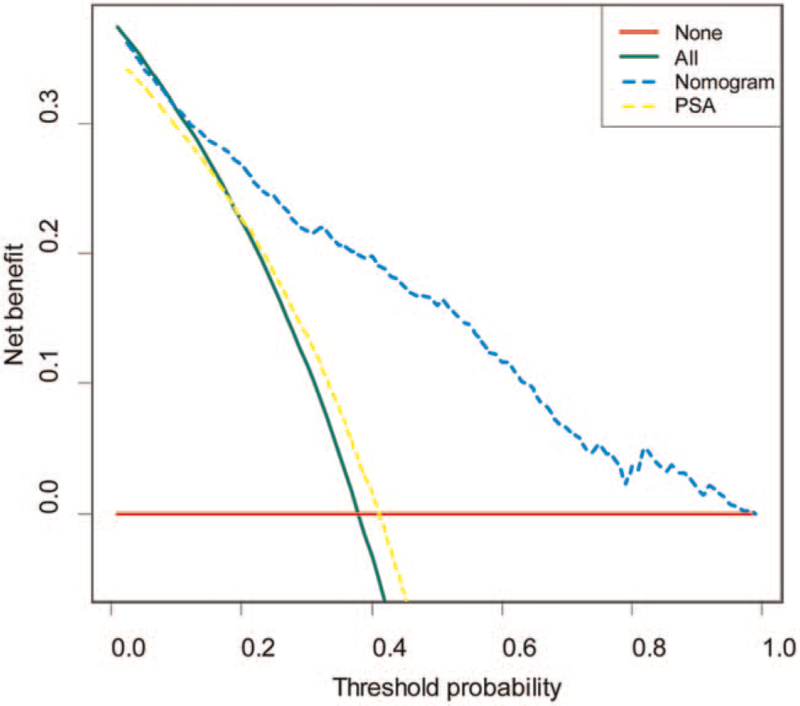
Decision curves analysis for the nomogram and the PSA for predicting the risk of PCa in clinical practice.

The potential clinical benefits of the 2 nomograms were also evaluated by decision curve analysis (Fig. 5). For predicting PCa, the nomogram was shown to achieve more benefits than the intervention-all-patients scheme, the intervention-none scheme, or PSA screening.

## 4 Discussion

It is always one of the research hotspots that how to improve the predictive accuracy before prostate biopsy. Nomogram is a simple graphical presentation of statistical methods, such as logistic regression and Cox proportional hazards analysis, which is popular in clinical risk or prognosis prediction. In the current study, we develop a nomogram for predicting the risk of PCa using 4 common clinical and laboratory variables: age, tPSA, fPSA, and prostate volume. All these factors could be noninvasively and easily obtained before the initial biopsy. And if a patient is without suspicious DRE or ultrasound but elevated PSA, we can also calculate the positive risk for prostate biopsy using his PSA value, age, and prostate volume. The area under ROC of our nomogram is 0.857, which is significantly higher than PSA (0.664), f/t PSA (0.669), and PSAD (0.801), respectively. Calibration plot and decision curve analysis validate the efficacy and potential benefits of our nomogram. This nomogram is used as a screening tool for prostate biopsy and should avoid missed diagnosis of PCa patients as much as possible. Therefore, we choose 95% sensitivity as the cut off value to ensure that the model has a low false-negative rate. Comparing with f/t PSA and PSAD, the nomogram has higher specificity and NPV, which could avoid 42.5% of unnecessary biopsies and 25.3% of total biopsies with missing only 4.4% (9 patients) of PCa patients. Among them, 3 patients’ gleason score are 6, 2 are 3 + 4 = 7, 2 are 4 + 3 = 7, 1 is 8, and 1 is 9.

Considering sextant biopsy, which was commonly performed in the past, is no longer adequate for detection of PCa, our study enrolled patients who underwent 12-core biopsy according to the current recommendation.^[23–25]^ Moreover, by description analysis of our cohort of patients, we found the positive rate of biopsy for patients with a tPSA level > 20 ng/mL reached 70%, while the rate for patients with a tPSA level 10 to 20 and < 10 ng/mL was only 46% and 32%, respectively. We also tested our nomogram on a larger group of 872 patients that contained patients with a tPSA level >20 ng/mL, and the AUC increased to 0.864 for this group. Although the result was more encouraging with the inclusion of patients with high tPSA values, we limited our included patients to the men with a tPSA level ≤ 20 ng/mL. The clinical usability and value would be certainly questioned and jeopardized with inclusion of patients with high tPSA levels, because most of them will undergo prostate biopsy in clinical practices. In addition, DRE and TRUS results were commonly reported to be risk factors for prostate biopsy, but they have poor sensitivity and high interobserver variability. Therefore, our nomogram was constructed without consideration of DRE and TRUS, which was performed by several different doctors.

Several nomograms have also been developed for predicting PCa before biopsy. Karakiewics et al^[26]^ developed a nomogram to estimate the risk of PCa, including age, PSA, f/t PSA, and DRE. However, the included patients had a PSA level ≤ 50 ng/mL, which weaken the usability and benefits of the model in routine clinical practices. Besides, all the enrolled patients had received sextant biopsy, which may underestimate the positive rate and is no longer recommended as the standard biopsy scheme. And Nguyen et al^[27]^ had proved that a previous risk model based on sextant biopsy scheme did not perform well in their cohort of men screened for PCa with extended prostate biopsy scheme.

Optenberg et al^[28]^ developed another nomogram for PCa enrolled 633 patients with a PSA level ≤ 20 ng/mL, but they did not take the free PSA into consideration. Free PSA is an effective tool to differentiate PCa from BPH, which is widely used in clinical practice. We also tested the contribution of fPSA to our nomogram. The inclusion of the fPSA increased the AUC of our nomogram from 0.816 to 0.857.

Eastham et al^[29]^ constructed a model based on a group of 700 patients with an AUC of 0.75. Although they just include the patients with a PSA level <4 ng/mL and with abnormal DRE, their model could not be applied in men with higher PSA level or normal DRE finding. Carlson et al^[30]^ included patients with a PSA range of 4 to 10 ng/mL, but they missed the group of patients with lower PSA level (< 4 ng/mL), which constituted significant 14% of our whole biopsy-positive population. More importantly, they did not report a definite AUC of their model.

Race is a significant risk factor for presentation of PCa. However, most nomograms were developed based on European or American population, for example, Zaytoun et al^[31]^ constructed a nomogram included the race factor. The genetic and environmental differences may preclude the transfer of existing nomograms from Western studies to Asian populations. Besides, few studies focused on Asian, especially Chinese, population. Zhu et al^[32]^ developed a prostate health index (PHI)-based nomogram for predicting PCa based on Chinese population. Their model showed high accuracy (0.839), which included age, prostate volume, and PHI. PHI was calculated using the following formula: (p2PSA/fPSA) × $\sqrt{PSA}$. However, the p2PSA had not been routinely applied in China, which limited its application significantly. Fang et al^[33]^ also constructed a nomogram based on Chinese population including MRI information, although the MRI parameters and interpretation criteria are not uniform among different hospitals, which also limits its widely application. In addition, they also included patients with a PSA level < 50 ng/mL, which may jeopardize the model's clinical usability. Another Korean study developed a nomogram that enrolled patients with a PSA level < 10 ng/mL; however, their model had not been internal or external validated.^[34]^

Several limitations still exist in our nomogram. The first one is that our study is a single-center retrospective analysis, and the selection bias may exist in our study and interfere with the accuracy of our model. And a future external validation would be required to evaluate the utility of our nomogram by using the data from other institutions or models. Second, the false-negative findings of the prostate biopsy were not taken into consideration in this study, because the data of repeated biopsy in our cohort are scarce. Finally, the MRI tests of our cohort were performed by several different hospitals before coming to our center, and the prostate imaging reporting and data system (PI-RADS) score was not indicated in the record. Considering the potential variability, we did not include the MRI result as a risk factor in our model.

In conclusion, we develop and validate a nomogram for predicting the risk of PCa at initial prostate biopsy in Chinese patients with PSA ≤ 20 ng/mL, which relies on 4 easily obtained factors, including age, tPSA, fPSA, and prostate volume. The nomogram provides a higher prediction accuracy than tPSA, f/t PSA, or PSAD without increase of medical cost or invasiveness, which could be used to avoid the unnecessary biopsies in clinical practice.

## Author contributions

**Conceptualization:** Xiaotao Yin, Jiangping Gao.

**Data curation:** Qiang Wu, Fanglong Li.

**Formal analysis:** Qiang Wu, Xiaotao Yin.

**Investigation:** Qiang Wu, Fanglong Li.

**Methodology:** Fanglong Li, Xiaotao Yin.

**Project administration:** Xiaotao Yin, Jiangping Gao.

**Supervision:** Xiaotao Yin.

**Writing – original draft:** Qiang Wu.

**Writing – review & editing:** Jiangping Gao, Xu Zhang.

## Correction

The corresponding author has been corrected from Xiaotao Yin to Jianping Gao.

## References

[1] Chen W Zheng R Baade PD. Cancer statistics in China, 2015. CA Cancer J Clin 2016;66:115–32. 26808342 10.3322/caac.21338

[2] Mottet N Bellmunt J Bolla M. EAU-ESTRO-SIOG Guidelines on Prostate Cancer. Part 1: screening, diagnosis, and local treatment with curative intent. Eur Urol 2017;71:618–29. 27568654 10.1016/j.eururo.2016.08.003

[3] Ilic D Djulbegovic M Jung JH. Prostate cancer screening with prostate-specific antigen (PSA) test a systematic review and meta-analysis. BMJ 2018;362:k3519. 30185521 10.1136/bmj.k3519 PMC6283370

[4] Cheng KC Lam WC Chan HC. Emergency attendances and hospitalisations for complications after transrectal ultrasound-guided prostate biopsies: a five-year retrospective multicentre study. Hong Kong Med J 2019;25:349–55. 31601774 10.12809/hkmj197825

[5] Huang GL Kang CH Lee WC Chiang PH. Comparisons of cancer detection rate and complications between transrectal and transperineal prostate biopsy approaches: a single center preliminary study. BMC Urol 2019;19:101. 31660936 10.1186/s12894-019-0539-4 PMC6816188

[6] Aminsharifi A Howard L Wu Y. Prostate specific antigen density as a predictor of clinically significant prostate cancer when the prostate specific antigen is in the diagnostic gray zone: defining the optimum cutoff point stratified by race and body mass index. J Urol 2018;200:758–66. 29758219 10.1016/j.juro.2018.05.016

[7] Catalona WJ Smith DS Ratliff TL. Measurement of prostate-specific antigen in serum as a screening test for prostate cancer. N Engl J Med 1991;324:1156–61. 1707140 10.1056/NEJM199104253241702

[8] Hendriks RJ van Oort IM Schalken JA. Blood-based and urinary prostate cancer biomarkers: a review and comparison of novel biomarkers for detection and treatment decisions. Prostate Cancer Prostatic Dis 2017;20:12–9. 27922627 10.1038/pcan.2016.59

[9] Chun FK Epstein JI Ficarra V. Optimizing performance and interpretation of prostate biopsy: a critical analysis of the literature. Eur Urol 2010;58:851–64. 20884114 10.1016/j.eururo.2010.08.041

[10] Ahyai SA Graefen M Steuber T. Contemporary prostate cancer prevalence among T1c biopsy-referred men with a prostate-specific antigen level < or = 4.0 ng per millilitre. Eur Urol 2008;53:750–7. 17964070 10.1016/j.eururo.2007.10.017

[11] Frånlund M Arnsrud Godtman R Carlsson SV. Prostate cancer risk assessment in men with an initial P.S.A below 3 ng/mL: results from the Göteborg randomized population-based prostate cancer screening trial. Scand J Urol 2018;52:256–62. 30241447 10.1080/21681805.2018.1508166 PMC6298808

[12] Kim JK Choi MJ Lee JS. A deep belief network and Dempster-Shafer-Based Multiclassifier for the pathology stage of prostate cancer. J Healthc Eng 2018;2018:4651582. 29755715 10.1155/2018/4651582 PMC5884161

[13] Eifler JB Feng Z Lin BM. An updated prostate cancer staging nomogram (Partin tables) based on cases from 2006 to. BJU Int 2013;111:22–9. 22834909 10.1111/j.1464-410X.2012.11324.x PMC3876476

[14] Hu X Cammann H Meyer HA. Artificial neural networks and prostate cancer--tools for diagnosis and management. Nat Rev Urol 2013;10:174–82. 23399728 10.1038/nrurol.2013.9

[15] Winter A Kneib T Wasylow C. Updated nomogram incorporating percentage of positive cores to predict probability of lymph node invasion in prostate cancer patients undergoing sentinel lymph node dissection. J Cancer 2017;8:2692–8. 28928857 10.7150/jca.20409 PMC5604200

[16] Elshafei A Chevli KK Moussa AS. PCA3-based nomogram for predicting prostate cancer and high grade cancer on initial transrectal guided biopsy. Prostate 2015;75:1951–7. 26384170 10.1002/pros.23096

[17] Cormio L Cindolo L Troiano F. Development and internal validation of novel nomograms based on benign prostatic obstruction-related parameters to predict the risk of prostate cancer at first prostate biopsy. Front Oncol 2018;8:438. 30386737 10.3389/fonc.2018.00438 PMC6198078

[18] Nan LB Yin XT Gao JP. Significant diagnostic value of free-serum PSA (FPSA)/Prostate-specific antigen density (PSAD) and (F/T)/PSAD for prostate cancer of the Chinese population in a single institution. Med Sci Monit 2019;25:8345–51. 31691648 10.12659/MSM.916900 PMC6859934

[19] Harrell FE Jr Lee KL Mark DB. Multivariable prognostic models: issues in developing models, evaluating assumptions and adequacy, and measuring and reducing errors. Stat Med 1996;15:361–87. 8668867 10.1002/(SICI)1097-0258(19960229)15:4<361::AID-SIM168>3.0.CO;2-4

[20] Robin X Turck N Hainard A. pROC: an open-source package for R and S+ to analyze and compare ROC curves. BMC Bioinform 2011;12:77. 10.1186/1471-2105-12-77 PMC3068975 21414208

[21] DeLong ER DeLong DM Clarke-Pearson DL. Comparing the areas under two or more correlated receiver operating characteristic curves: a nonparametric approach. Biometrics 1988;44:837–45. 3203132

[22] Vickers AJ Cronin AM Elkin EB Gonen M. Extensions to decision curve analysis, a novel method for evaluating diagnostic tests, prediction models and molecular markers. BMC Med Inform Decis Mak 2008;8:53. 19036144 10.1186/1472-6947-8-53 PMC2611975

[23] Bjurlin MA Carter HB Schellhammer P. Optimization of initial prostate biopsy in clinical practice: sampling, labeling and specimen processing. J Urol 2013;189:2039–46. 23485507 10.1016/j.juro.2013.02.072 PMC3925148

[24] Chen Y Jiang X Liu R. The specific choice of transrectal ultrasound-guided prostate biopsy scheme based on prostate specific antigen and prostate specific antigen density. Med Sci Monit 2019;25:6230–5. 31424055 10.12659/MSM.915826 PMC6752102

[25] Mohler JL Antonarakis ES Armstrong AJ. Prostate Cancer, Version 2.2019, NCCN Clinical Practice Guidelines in Oncology. J Natl Compr Canc Netw 2019;17:479–505. 31085757 10.6004/jnccn.2019.0023

[26] Karakiewicz PI Benayoun S Kattan MW. Development and validation of a nomogram predicting the outcome of prostate biopsy based on patient age, digital rectal examination and serum prostate specific antigen. J Urol 2005;173:1930–4. 15879784 10.1097/01.ju.0000158039.94467.5d PMC1855288

[27] Nguyen CT Yu C Moussa A. Performance of prostate cancer prevention trial risk calculator in a contemporary cohort screened for prostate cancer and diagnosed by extended prostate biopsy. J Urol 2010;183:529–33. 20006887 10.1016/j.juro.2009.10.007

[28] Optenberg SA Clark JY Brawer MK. Development of a decision-making tool to predict risk of prostate cancer: the Cancer of the Prostate Risk Index (CAPRI) test. Urology 1997;50:665–72. 9372872 10.1016/S0090-4295(97)00451-2

[29] Eastham JA May R Robertson JL. Development of a nomogram that predicts the probability of a positive prostate biopsy in men with an abnormal digital rectal examination and a prostate-specific antigen between 0 and 4 ng/mL. Urology 1999;54:709–13. 10510933 10.1016/s0090-4295(99)00213-7

[30] Carlson GD Calvanese CB Partin AW. An algorithm combining age, total prostate-specific antigen (PSA), and percent free PSA to predict prostate cancer: results on 4298 cases. Urology 1998;52:455–61. 9730460 10.1016/s0090-4295(98)00205-2

[31] Zaytoun OM Kattan MW Moussa AS. Development of improved nomogram for prediction of outcome of initial prostate biopsy using readily available clinical information. Urology 2011;78:392–8. 21705045 10.1016/j.urology.2011.04.042

[32] Zhu Y Han CT Zhang GM. Development and external validation of a prostate health index-based nomogram for predicting prostate cancer. Sci Rep 2015;5:15341. 26471350 10.1038/srep15341 PMC4607975

[33] Fang D Zhao C Ren D. Could magnetic resonance imaging help to identify the presence of prostate cancer before initial biopsy? The development of nomogram predicting the outcomes of prostate biopsy in the Chinese Population. Ann Surg Oncol 2016;23:4284–92. 27464612 10.1245/s10434-016-5438-2

[34] Ahn JH Lee JZ Chung MK Ha HK. Nomogram for prediction of prostate cancer with serum prostate specific antigen less than 10 ng/mL. J Korean Med Sci 2014;29:338–42. 24616581 10.3346/jkms.2014.29.3.338 PMC3945127

